# Role of CD4^+^ T Cells in Allergic Airway Diseases: Learning from Murine Models

**DOI:** 10.3390/ijms21207480

**Published:** 2020-10-11

**Authors:** Kento Miura, Kimiko Inoue, Atsuo Ogura, Osamu Kaminuma

**Affiliations:** 1 Department of Disease Model, Research Institute of Radiation Biology and Medicine, Hiroshima University, Hiroshima 734-8553, Japan; kmiura@hiroshima-u.ac.jp; 2 RIKEN BioResource Research Center, Ibaraki 305-0074, Japan; inoue@rtc.riken.go.jp (K.I.); ogura@rtc.riken.go.jp (A.O.)

**Keywords:** allergy, airway inflammation, CD4^+^ T cell, somatic cell nuclear transfer, T-cell receptor

## Abstract

The essential contribution of CD4^+^ T cells in allergic airway diseases has been demonstrated, especially by using various murine models of antigen-induced airway inflammation. In addition to antigen-immunized mouse models employing mast cell-deficient mice and CD4^+^ T cell-depleting procedure, antigen-specific CD4^+^ T cell transfer models have revealed the possible development of allergic inflammation solely dependent on CD4^+^ T cells. Regardless of the classical Th1/Th2 theory, various helper T cell subsets have the potential to induce different types of allergic inflammation. T cell receptor (TCR)-transgenic (Tg) mice have been used for investigating T cell-mediated immune responses. Besides, we have recently generated cloned mice from antigen-specific CD4^+^ T cells through somatic cell nuclear transfer. In contrast to TCR-Tg mice that express artificially introduced TCR, the cloned mice express endogenously regulated antigen-specific TCR. Upon antigen exposure, the mite antigen-reactive T cell-cloned mice displayed strong airway inflammation accompanied by bronchial hyperresponsiveness in a short time period. Antigen-specific CD4^+^ T cell-cloned mice are expected to be useful for investigating the detailed role of CD4^+^ T cells in various allergic diseases and for evaluating novel anti-allergic drugs.

## 1. Introduction

**Figure 1:**
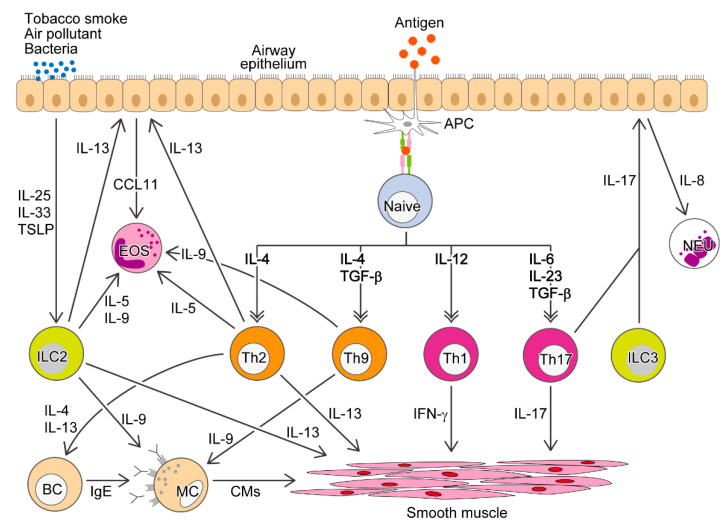
Key players in the development of allergic airway inflammation. Foreign antigens exposed to airway epithelium are processed and presented by APCs to unprimed naive CD4^+^ T cells. Then the naive CD4^+^ T cells differentiate into various Th cell subsets, largely depending on their respective cytokines in the surrounding environment. Individual Th subsets produce their characteristic cytokines and contribute to the development of allergic airway inflammation. The involvement of new lymphoid cells such as group 2 innate lymphoid cells (ILC2) and ILC3 has recently been reported. BC, B cell; EOS, eosinophil; CMs, chemical mediators; MC, mast cell; NEU, neutrophil; TSLP, thymic stromal lymphopoietin.

Antigen-specific CD4^+^ cells play a central role in the acquired immune system. After differentiation and maturation in the thymus, unprimed naive CD4^+^ T cells are distributed in peripheral tissues. Upon recognition of a foreign antigen with antigen-presenting cells (APCs), CD4^+^ T cells differentiate into various helper T (Th) cell subsets such as Th1 and Th2 according to the surrounding environment including their responsible cytokines [1,2]. Among various cells and soluble factors, Th subsets and their signature cytokines are individually or collaboratively implicated in various immune disorders including allergic diseases (Figure 1). A variety of murine models have been developed and utilized to investigate the contribution of CD4^+^ T cells to allergic inflammation. In this review, we describe the current knowledge about the role of CD4^+^ T cells in the pathogenesis of allergic airway diseases mainly revealed by employing their murine models. Furthermore, we introduce antigen-specific T cell-cloned mice as new useful animals for investigating allergic airway inflammation.

## 2. Role of CD4^+^ T Cells in Allergic Inflammation

In the latter half of 1980s, pathogenetic understanding of allergic diseases advanced drastically due to the establishment of pathological investigation methods, such as in situ hybridization and immunostaining. Numerous evidences indicating the importance of CD4^+^ T cells and T cell cytokines for the development of inflammatory responses in allergic diseases were accumulated in the early 1990s. Activated CD4^+^ T cells were found to be abundant in bronchial mucosa of patients with chronic asthma and in skin tissue of atopic dermatitis patients [3,4]. Moreover, the expression of various cytokines in CD4^+^ T cells was augmented upon antigenic challenge [5] and it was correlated with local eosinophil infiltration [6]. T cell cytokine levels in bronchoalveolar lavage fluid (BALF) were elevated in both atopic and non-atopic bronchial asthma patients [7]. T cell cytokine concentrations were also elevated in the blood of asthmatics, whereas the concentrations were decreased in parallel with symptomatic remission following steroid treatment [8]. In antigen-immunized murine models of allergic airway inflammation, the infiltration of eosinophils induced by local antigen exposure was strongly suppressed by the elimination of CD4^+^ T cells [9]. These data suggest that activation of CD4^+^ T cells may be a predisposing factor for the development of asthma. 

## 3. IgE/Mast Cell- and CD4^+^ T Cell-Dependent Cascades in Allergic Inflammation

Since the discovery of immunoglobulin (IgE) by Ishizaka et al. in 1966 [10], it has been widely recognized that the activation of mast cells via IgE cross-linkage directly causes various allergic symptoms. This IgE/mast cell-mediated pathway is substantially implicated in the pathogenesis of allergic inflammation. A humanized anti-IgE antibody, omalizumab, was approved for treating allergic diseases including asthma, urticaria, and rhinitis [11,12,13,14,15]. The IgE/mast cell-mediated responses are closely related with T cell-dependent mechanisms in the development of allergic inflammation. IgE is not produced in T cell-deficient nude mice [16], in which thymus development is impaired due to *Foxn1* mutation [17]. Mast cells contribute to the activation of T cells through antigen presentation and production of TNF-α and IL-6 [18]. 

To investigate the role of mast cells in the development of allergic eosinophilic inflammation, many studies employing mast cell-deficient WBB6F1-*W*/*W^v^* mice (*W*/*W^v^*) [19] were performed in the 1990s, though the findings were controversial. The critical contribution of mast cells was demonstrated in some groups [20,21], whereas others appealed their dispensability [22]. To solve the confusion, a time course study of antigen-induced airway inflammation was performed. Airway eosinophil infiltration induced by antigen challenge in immunized *W*/*W^v^* mice was about half of that in wild-type mice 48 h after the challenge, whereas almost the same degree of eosinophil infiltration was observed in both groups at 6, 24, 96, and 216 h, respectively [23]. This suggests that mast cells were responsible for half of the eosinophil infiltration 48 h after the challenge, while the other half of infiltration at 48 h and almost all at other time points was not mediated by mast cells (Table 1). 

To investigate the contribution of CD4^+^ T cells to mast cell-independent eosinophil infiltration, immunized mice were treated with an anti-CD4 antibody prior to antigen exposure. Along with the potent depletion of peripheral CD4^+^ T cells, the infiltration of eosinophils observed in *W*/*W^v^* mice after 48 and 96 h of the challenge largely disappeared. By conducting the same treatment in immunized normal littermate mice, the infiltration of eosinophils was also found to be reduced to approximately half at 48 h, and almost completely suppressed at 96 h [23]. It was suggested that the development of allergic eosinophilic inflammation, largely depended on CD4^+^ T cells, while IgE/mast cells were only partly involved (Table 1). Using CD4^+^ T cell-depleting procedure, the essential contribution of CD4^+^ T cells were also demonstrated by other researchers [9,24] and in other allergy models [11,25].

Particularly related with allergic skin diseases, Mukai et al. reported the possible development of IgE- but not mast cell- or T cell-dependent eosinophilic skin inflammation in murine models [26]. Delayed-type ear swelling with eosinophil infiltration was induced in anti-2,4,6-trinitrophenol (TNP)-IgE transgenic (Tg) mice upon challenge with TNP-conjugated ovalbumin. They discovered that these responses were almost completely dependent on basophils. Interestingly, the crucial contribution of basophils to allergic airway inflammation was hardly observed. Antigen-induced lung accumulation of eosinophils in immunized mice was weakly suppressed, though bronchial hyperresponsiveness (BHR) was not affected, by depleting basophils [27]. Obvious eosinophil accumulation in the nasal mucosa was observed in antigen-immunized wild-type mice but not in non-immunized anti-ovalbumin IgE-Tg mice even upon challenge with the corresponding antigen [28]. It is suggested that the existence of IgE, despite its mast cell and basophil activating property, is insufficient for the development of airway eosinophilic inflammation (Table 1). 

Nevertheless, considering the clinical effectiveness of omalizumab, it was unlikely that asthma-like airway inflammation could be reproduced even in mouse models without antigen-specific IgE. To confirm this and evaluate the CD4^+^ T cell-dependent reaction alone, novel mouse models were developed [29,30]. A number of antigen-specific T cells were prepared by cultivating splenic CD4^+^ T cells of immunized mice in the presence of the corresponding antigen and APCs in vitro, then they were adoptively transferred to non-immunized wild-type mice. Surprisingly, asthma-like airway eosinophilic inflammation, accompanied by BHR, was reproduced in those mice upon inhalation of the challenge with the corresponding antigen [29]. Regardless of the lack of enough time to produce antigen-specific IgE in this procedure, the airway inflammation induced in antigen-specific T cell-transferred mice was comparable to the response observed in antigen-immunized and -challenged mice. Therefore, CD4^+^ T cells have potential to elicit allergic airway inflammation without assistance of the IgE/mast cell-mediated pathway (Table 1). Recently, CD4^+^ T cell-dependent allergic inflammation models are widely used and applied for investigating allergic diseases not only in the respiratory tract but also in other target organs, such as nasal mucosa, skin, and the digestive tract [31,32,33]. These findings suggest that antigen-specific CD4^+^ T cells are universally responsible for allergic diseases in various organs.

## 4. T Cell Subsets and Allergic Airway Diseases

Concerning the relationship between T cell subsets and allergic airway diseases, the Th1/Th2 theory proposed by Mossman et al. [2,34,35] in the 1980s largely influenced the understanding of disease pathogenesis and strategies for their treatments. The theory explains that allergic airway diseases associated with eosinophilic inflammation develop due to the dominant differentiation of Th2 cell subset, which preferentially produces IL-4, -5, and -13 rather than Th1 cell subset, which produces IFN-γ and TNF-〈 [36,37,38,39,40,41,42]. Increase in Th2 cytokine levels and the number of Th2 cytokine-expressing T cells are observed in the bronchial mucosa of asthmatic patients [3,5,43,44,45]. Among Th2 cytokines, we found that human peripheral CD4^+^ T cells in both atopic and non-atopic asthmatics exhibited increased IL-5-producing capacity [46]. The in vitro IL-5-producing ability of various antigen-specific T cell clones correlated with the degree of in vivo antigen-induced airway eosinophil infiltration that developed in T cell-transferred mice [47]. Furthermore, T cell-mediated eosinophilic inflammation was suppressed by anti-IL-5 antibody treatment [29], suggesting that Th2 cell subset plays a crucial role in the development of allergic airway diseases, especially via the production of IL-5. Although the contribution of eosinophils to BHR was sometimes controversial in conventional antigen-immunized models [48,49], antigen-induced BHR developed in Th2 cell-transferred mice disappeared when eosinophil-deficient mice were used as recipients [50]. Consistently, anti-IL-5 (mepolizmab) and anti-IL-5 receptor 〈 (benralizmab) antibodies are frequently used to treat bronchial asthma accompanied by obvious eosinophilic inflammation [29].

Until the latter half of the 1990s, the Th1/Th2 theory in allergic diseases was supported by the improvement of airway inflammation in murine models of asthma by introduction of *Ifnγ* gene or transfer of Th1 cells [51,52]. However, since Hansen et al. reported in 1999 that antigen-induced BHR developed even in Th1 cell-transferred mice [53], many investigations have demonstrated that not only Th2 cell-mediated eosinophilic inflammation, but also other Th subset-dependent mechanisms are involved in the development of allergic diseases (Figure 1, Table 2). The existence of various T cell subsets, such as Th9 [54,55,56], Th17 [57,58,59], Th22 [60], Th25 [61], and Th31 [62], has been proposed. The differentiation into individual T cell subsets and production of their characteristic cytokines are associated with epigenetic regulation such as DNA methylation and histone modifications [63,64].

Among various Th subset, Th9 and Th17 cells have been proven to induce airway inflammation accompanied by significant BHR in mice via adoptive transfer (Table 2) [65,66,67]. The airway inflammation mediated by Th17 cells, as well as Th1 cells, was characterized by massive accumulation of neutrophils [65,66]. On the other hand, Th9 cells, like Th2 cells, have potential to induce eosinophil-dominant airway inflammation [50,67], though Th2- but not Th9-mediated BHR was dependent on eosinophils [50]. Interestingly, antigen-induced BHR developed in mice transferred with Th2, but not Th9 or Th17 cells, was suppressed by systemic administration of dexamethasone [65,67], suggesting that Th9 and Th17 cells play a role in the development of steroid-resistant asthma (Table 2). At least in some of the patients with bronchial asthma, the accumulation of Th1 or Th17 cells into the respiratory tract [68,69] and increase in Th9 cells and IL-9 concentration in peripheral blood [70,71] were observed. Significant correlation between serum IL-17 level and clinical severity was reported in allergic rhinitis patients [72]. The pathogenesis independently of the Th2/IL-5/eosinophil-axis may be involved in those patients (non-Th2 asthma). Mouse models of typical Th2, non-Th2, and their mixed type airway inflammation has recently been developed by employing procedures with different route and dose of antigen administration and with the usage of lipopolysaccharide [73,74]. 

Regulatory T cell (Treg), a non-helper T cell subset, plays a crucial role in antigen tolerance induction [75]. Possible involvement of Tregs in allergic airway diseases has been suggested by their suppressive activity against various cell types including T cells, eosinophils, and mast cells [76]. Beyond T cell subsets, the contribution of new lymphoid cells such as group 2 innate lymphoid cells (ILC2), which do not have antigen specificity and produce IL-5, IL-9, and IL-13, and ILC3, which produce IL-17, has recently been reported [77,78,79,80,81] (Figure 1). Due to the appearance of these cytokine-producing non-T cells, the type 2/non-type 2 classification is becoming more popular than Th2/non-Th2. The relative participation of these T cells and non-T cells in the pathogenesis of allergic airway inflammation deserves further investigation.

Based on new evidence showing the difference in the processes leading to the disease conditions and in their responsible Th and other cells among patients with allergic airway diseases, a new concept for developing an appropriate treatment according to the endotype of each patient has recently been proposed [82,83]. 

## 5. Antigen-Specific T Cell Receptor Tg Mice

Tg mice expressing an antigen-specific T cell receptor (TCR), which is composed of α and β chains, are frequently used to examine T cell-mediated immune responses [84]. DO11.10, a typical TCR-Tg mouse, expresses TCR that recognizes a peptide sequence derived from ovalbumin (OVA323-339) [85]. In part of the T cell-transfer models, DO11.10 mice were employed to prepare antigen-specific CD4^+^ T cells as donor cells [50,65,66,67,86,87]. OVA23-3 is another TCR-Tg mouse expressing OVA323-339-reactive TCR [88]. Interestingly, OVA23-3 mice developed food allergy-like intestinal inflammation with Th2-favoured responses upon feeding with an egg white diet [89,90], whereas DO11.10 mice were used for a tolerance induction model showing Th1-phenotype [91]. Tg mice expressing mite antigen-reactive TCR generated by Jarman et al. developed weak airway inflammation following repeated intratracheal antigen instillation [92]. Surprisingly, Th1/Th17 dominant airway inflammation is induced in DO11.10 mice with BALB/c-background upon antigen challenge [93], although BALB/c is known to be a Th2-favoured inbred strain.

The differential and unexpected phenotypes of TCR-Tg mouse lines are probably caused, at least in part, by TCR artificially introduced in the mouse genome. The antigen reactivity of T cells as well as the differentiation into distinct Th cell subsets is largely affected by the amount and quality of antigen stimulation received through TCR. Hosken et al. and Constant et al. demonstrated that weak TCR stimulation with low antigen concentration causes Th2 differentiation, whereas strong stimulation promotes Th1 differentiation in vitro [94,95]. Therefore, the Th1/Th17 dominant airway inflammation observed in DO11.10 mice [93] might be induced by stronger activation of T cells through the artificially introduced TCR. Most importantly, the expression pattern of the antigen-specific TCR and resulting antigen reactivity in the Tg mouse T cells are, at least in some cases, likely to differ from those in wild-type mouse T cells.

Among patients with allergic diseases, the antigen reactivity, as well as kinds of reactive antigens, is also extremely different. However, these differences are hardly explained by the diversity of TCR, because the germline TCR locus is highly conserved. Since major histocompatibility complex (MHC) haplotype shows extensive diversity [96], stimulation by a certain antigen epitope through the distinct MHC leads to different responses of T cells in individual patients, including their subset differentiation. This may be one of the reasons for the existence of various disease phenotypes and endotypes in allergic airway diseases. 

## 6. Generation of Cloned Mice Derived from Antigen-Specific CD4^+^ T Cells 

To further investigate CD4^+^ T cell-dependent allergic inflammation beyond the limits of TCR-Tg mice as described above, we generated a new mouse model containing T cells that express endogenously regulated antigen-specific TCR via somatic cell nuclear transfer technology. Somatic cell-cloned animals, which came into the limelight with the birth of “Dolly”, the cloned sheep, in 1997 [97], are animals generated from somatic cells that had differentiated into specific tissues, such as skin and other organs. They are generally produced by transferring a somatic cell nucleus into an enucleated oocyte of another individual, then the reconstructed embryos are transplanted into foster mothers. In the resulting born animals, the genetic information of the donor somatic cell is conserved.

In typical murine allergy models, production of antigen-specific CD4^+^ T cells is induced in normal mice by the systemic administration of an antigen with an adjuvant. In response to subsequent antigen exposure, mice exhibit organ-specific allergic inflammation in vivo. The antigen-specific CD4^+^ T cells that develop in the immunized mice, in which *Tcrα* and *Tcrβ* genes are optimally rearranged, express a functional TCR. If cloned mice inheriting the genetic information of the rearranged TCR are generated, it is expected that the antigen-specific TCR would be expressed in all the mature T cells in their body.

However, there were technical problems associated with producing cloned mice from antigen-specific T cells. Since the birth of the first cloned mouse was revealed by Wakayama et al. in 1998 [98], cloned mice have been produced from various somatic cells, including those of T-cell lineages. The first T cell-originated monoclonal mouse was generated from a peripheral T cell [99,100]. T cells of the cloned mice were identified as MHC class I-restricted, though their reactive antigen was not clarified. Cloned mice were also generated using freshly isolated antigen-specific CD8^+^ T cells [101]. We found that the developmental potential of a characteristic T-cell population, natural killer T (NKT) cell, was unexpectedly high, and that live cloned fetuses could be obtained from purified liver NKT cells [102]. Several technical improvements, including the use of histone deacetylase inhibitors to treat reconstructed embryos, have also enabled cloning from peripheral blood lymphocytes [103]. 

**Figure 2:**
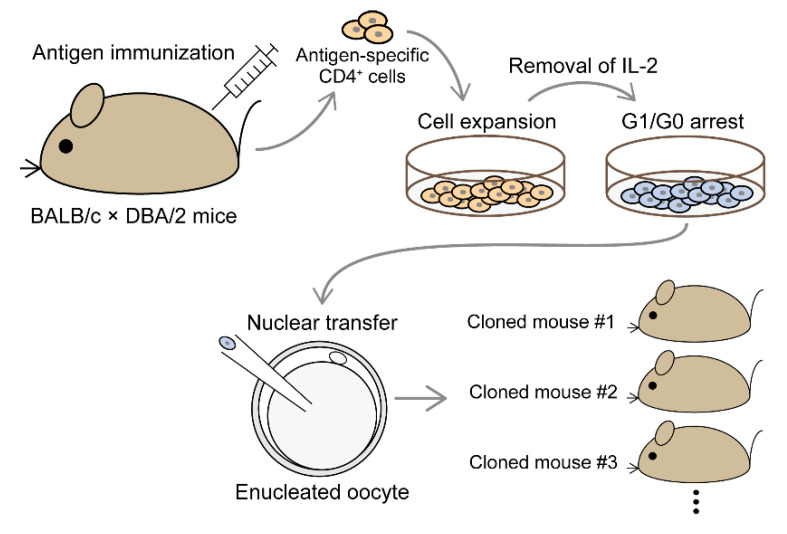
Schematic procedure for generating cloned mice from antigen-specific CD4^+^ T cells. Antigen-specific CD4^+^ T cells obtained from immunized BALB/c × DBA/2 mice were expanded by antigen stimulation culture in vitro. Upon removal of IL-2, G1/G0 phase-synchronized cells were used for somatic cell nuclear transfer. Various lines of cloned mice were obtained.

Nevertheless, until about 20 years after the birth of the first cloned mouse, cloned mice derived from antigen-specific CD4^+^ T cells had not been produced. A major obstacle was that cells suitable for nuclear transfer cloning were limited to non-proliferating cells. The cell cycle of donor T cells is required to be in the G1/G0 phase to synchronize with that of recipient oocytes. Nonetheless, antigen-specific CD4^+^ T cells are usually obtained by in vitro stimulation culture of T cells from antigen-immunized animals in the presence of antigen and APCs. Thereafter, the cell cycle of antigen-reactive CD4^+^ T cells progresses asynchronously during the culture period. Therefore, it appeared to be difficult to prepare G1/G0 phase-synchronized antigen-specific T cells. This may have been one of the reasons why the production of cloned mice derived from antigen-specific CD4^+^ T cells had been unsuccessful. To overcome this problem, T cells grown by stimulation culture were once recovered, then they were further cultured for a few more days in a medium without growth factors, such as IL-2 (Figure 2). In addition, because donor cells from mice of mixed backgrounds are much more efficient than those from inbred mice for somatic cell nuclear transfer (SCNT) [104,105], and because MHC class I and II haplotypes in BALB/c are identical to those in DBA2 mice, donor CD4^+^ T cells were prepared from antigen-immunized male (BALB/c × DBA/2) F1 (CDF1) mice. These modifications allowed us to obtain more antigen-specific CD4^+^ T cells in the stationary phase, and to produce cloned mice from them [106] (Figure 2). 

## 7. Antigen Reactivity of CD4^+^ T Cells in Cloned Mice

Cloned mice from CD4^+^ T cells reactive to various antigens were successfully generated (Table 3): OVA323-339 same as the epitope of DO11.10 T cells; and two major mite antigens, *Dermatophagoides farinae* (*Der f*) and *D. pteronyssinus* (*Der p*). When the antigen reactivity was examined using the peripheral lymphocytes of the cloned mice, the cloned mice showed a proliferative response against the reactive antigen of the CD4^+^ T cells used as nuclear transfer donors. CD4^+^ T cells in these cloned mice expressed a pair of rearranged TCRα and β chains. Interestingly, the TCRα and β were identical in several cloned mice produced from *Der p*-reactive CD4^+^ T cells. It is likely that these mice were born from T cells originating from a single cell proliferated by antigen stimulation in vitro. 

The rearranged TCRα and β alleles of the cloned mice were inherited by their offspring upon mating with wild-type BALB/c mice according to Mendel’s law. Only CD4^+^ T cells of the F1 mice that had both rearranged TCRα and β alleles proliferated significantly upon stimulation with the corresponding antigen in vitro. These findings suggest that the heterodimerization of the arranged TCRα and β chains in T cells is required for their in vitro antigen reactivity (Table 3). Furthermore, CD4^+^ T cells, which proliferated in response to the antigen, showed different cytokine producing patterns among the cloned mouse strains. Since stimulation intensity of TCR affects the subset differentiation of CD4^+^ T cells as described above [94,95,107], it was suggested that various cloned mice containing TCRs with different binding intensity to the antigen/MHC complex were established. 

## 8. Usefulness of Antigen-Specific T Cell-Cloned Mice for Investigating Allergic Airway Inflammation In Vivo

Non-immunized wild-type mice required exposure to mite antigen more than a few dozen times over the course of a month to induce asthma-like airway inflammation [108,109,110]. However, strong airway inflammation with eosinophilic infiltration and BHR was developed in the cloned mice generated from *Der f*-reactive CD4^+^ T cells upon only four incidences of antigen exposure. OVA323-339-reactive cloned mice developed obvious nasal inflammation following 3 to 5 incidences of intranasal OVA challenge. Similar but weaker nasal inflammatory responses were induced in DO11.10 mice, whereas stronger responses were observed in wild-type mice transferred with CD4^+^ T cells of the cloned mice. Moreover, in this in vivo experimental system, unlike the in vitro antigen reactivity mentioned above, the cloned mice expressing both or either antigen-specific rearranged TCR〈 and ® chains displayed significantly stronger airway inflammation compared with wild-type mice (Table 3).

These nuclear transfer cloned mouse strains exhibited diverse phenotypes, although they were generated from CD4^+^ T cells reactive to the same antigen. For example, two strains of cloned mice (Df#1 and Df#2) generated from *Der f*-reactive T cells showed differences in thymus and peripheral CD4/CD8 ratios, as well as in the pattern of antigen-induced cytokine production. CD4^+^ T cells of Df#1 preferentially produced Th1 and Th17 cytokines, whereas those of Df#2 produced large amounts of Th2 cytokines upon antigen stimulation. Based on these in vitro findings, it was assumed that the in vivo allergic phenotype of Df#1 was close to that of DO11.10 mice represented by Th1/ Th17 dominant airway inflammation as described above. However, large amounts of IL-4, IL-5, and IL-13, and massive infiltration of eosinophils were detected in the BALF of Df#1 after antigen exposure, whereas almost no production of IFN-γ and IL-17 was observed. In contrast, eosinophil-less airway inflammation developed in Df#2, notwithstanding its in vitro Th2 dominant feature. Therefore, the subset differentiation property of peripheral T cells of the cloned mice may differ between in vitro and in vivo conditions. 

## 9. Conclusions

In this review, we described the role of CD4^+^ T cells in allergic airway diseases indicated by various mouse models. Upon differentiation into various Th cell subsets, CD4^+^ T cells contribute to diverse phenotypes and endotypes of allergic inflammation. Furthermore, we revealed that stable allergic airway inflammation could be easily induced in our cloned mice derived from antigen-specific CD4^+^ T cells. Since it is becoming clear that the cloned mice produce antigen-specific IgE in parallel with the onset of airway inflammation after only a few airway antigen challenges (unpublished data), the usefulness of the cloned mice could be extended to include analyzing the interaction between the T cell and IgE/mast cell cascades. TCRs expressed in the cloned mice are expected to differ in expression level and behavior from those in TCR-Tg mice. Therefore, some of the immunological theories substantiated by studies with TCR-Tg mice may need revision, by comparing the features of cloned mice and TCR-Tg mice. In these cloned mice, not only asthma-like airway inflammation, but also phenotypes that mimic various allergic diseases, such as allergic rhinitis, atopic dermatitis, and gastrointestinal allergies are now able to be reproduced. These new models of CD4^+^ T cell-mediated allergic inflammation using the cloned mice exert their full potential for elucidating the pathogenesis of allergic diseases and evaluating the efficacy and/or screening of anti-allergic drugs.

## Figures and Tables

**Table 1:** Animal models showing involvement of IgE/mast cells and CD4^+^ T cells in allergic airway inflammation.

Antigen-Challenged Animals	Antigen-Specific IgE/mast Cells	Antigen-Specific CD4^+^ T Cells	Eosinophil Infiltration
Non-immunized IgE-Tg	Yes	No	No
Immunized wild-type	Yes	Yes	Yes
Immunized *W*/*W^v^*	No	Yes	Yes
Immunized wild-type + anti-CD4 antibody	Yes	No	No
Immunized *W*/*W^v^* + anti-CD4 antibody	No	No	No
Non-immunized wild-type + antigen-specific CD4^+^ T cells	No	Yes	Yes

Significant induction of antigen-induced airway eosinophil infiltration was reported in immunized wild-type and *W*/*W^v^* mice and non-immunized wild-type mice transferred with antigen-specific CD4^+^ T cells but not in non-immunized anti-ovalbumin immunoglobulin (Ig) E-Tg mice. The depletion of CD4^+^ T cells by anti-CD4 antibody treatment diminished the eosinophil accumulation both in immunized wild-type and *W*/*W^v^* mice, suggesting the essential contribution of CD4^+^ T cells to allergic airway inflammation.

**Table 2:** Different features of Th cell subset-induced airway inflammation.

Features	Th2	Th9	Th1	Th17
Requirement of IgE/mast cells	No	No	No	No
BHR	Yes	Yes	Yes	Yes
Eosinophilia	Yes	Yes	No	No
Contribution of eosinophils of BHR	Yes	No	No	No
Steroid resistance	No	Yes	No	Yes

Among Th subsets, Th1, Th2, Th9, and Th17 cells have been reported to elicit antigen-induced bronchial hyperresponsiveness (BHR) in normal mice by adoptive transfer without assistance of IgE/mast cells. The typical eosinophilic inflammation was induced by both Th2 and Th9 cells, though only Th2-mediated BHR depended on eosinophils. The BHR mediated by Th9 and Th17 cells showed steroid resistance.

**Table 3:** Antigen-reactivity with the reconstituted TCR〈 and/or ® chains in the cloned mice.

Reactive Antigen	No. of Coned Mice	Requirement of Reconstituted TCRα and/or β for Antigen Reactivity			
		In Vitro		In Vivo	
		Both rα and rβ	Either rα or rβ	Both rα and rβ	Either rα or rβ
OVA323-339	1	Yes	N.T.	Yes	Yes
*Der f*	2	Yes	No	Yes	Yes
*Der p*	5	Yes	No	N.T.	N.T.

Only the offspring of cloned mice that had both reconstituted TCRα (rα) and β (rβ) alleles showed in vitro antigen reactivity. In contrast, in the in vivo experiments, those expressing either rα or rβ allele also displayed antigen reactivity. N.T.: not tested.

## References

[1] Tada T., Takemori T., Okumura K., Nonaka M., Tokuhisa T. (1978). Two distinct types of helper T cells involved in the secondary antibody response: Independent and synergistic effects of Ia- and Ia+ helper T cells. J. Exp. Med..

[2] Mosmann T.R., Cherwinski H., Bond M.W., Giedlin M.A., Coffman R.L. (1986). Two types of murine helper T cell clone. I. Definition according to profiles of lymphokine activities and secreted proteins. J. Immunol..

[3] Hamid Q., Azzawi M., Ying S., Moqbel R., Wardlaw A.J., Corrigan C.J., Bradley B., Durham S.R., Collins J.V., Jeffery P.K. (1991). Expression of mRNA for interleukin-5 in mucosal bronchial biopsies from asthma. J. Clin. Investig..

[4] Kay A.B., Ying S., Varney V., Gaga M., Durham S.R., Moqbel R., Wardlaw A.J., Hamid Q. (1991). Messenger RNA expression of the cytokine gene cluster, interleukin 3 (IL-3), IL-4, IL-5, and granulocyte/macrophage colony-stimulating factor, in allergen-induced late-phase cutaneous reactions in atopic subjects. J. Exp. Med..

[5] Robinson D., Hamid Q., Bentley A., Ying S., Kay A.B., Durham S.R. (1993). Activation of CD4+ T cells, increased TH2-type cytokine mRNA expression, and eosinophil recruitment in bronchoalveolar lavage after allergen inhalation challenge in patients with atopic asthma. J. Allergy Clin. Immunol..

[6] Krishnaswamy G., Liu M.C., Su S.N., Kumai M., Xiao H.Q., Marsh D.G., Huang S.K. (1993). Analysis of cytokine transcripts in the bronchoalveolar lavage cells of patients with asthma. Am. J. Respir. Cell Mol. Biol..

[7] Walker C., Virchow J.C., Bruijnzeel P.L., Blaser K. (1991). T cell subsets and their soluble products regulate eosinophilia in allergic and nonallergic asthma. J. Immunol..

[8] Corrigan C.J., Haczku A., Gemou-Engesaeth V., Doi S., Kikuchi Y., Takatsu K., Durham S.R., Kay A.B. (1993). CD4 T-lymphocyte activation in asthma is accompanied by increased serum concentrations of interleukin-5. Effect of glucocorticoid therapy. Am. Rev. Respir. Dis..

[9] Nakajima H., Iwamoto I., Tomoe S., Matsumura R., Tomioka H., Takatsu K., Yoshida S. (1992). CD4+ T-lymphocytes and interleukin-5 mediate antigen-induced eosinophil infiltration into the mouse trachea. Am. Rev. Respir. Dis..

[10] Ishizaka K., Ishizaka T., Hornbrook M.M. (1966). Physicochemical properties of reaginic antibody. V. Correlation of reaginic activity wth gamma-E-globulin antibody. J. Immunol..

[11] Milgrom H., Berger W., Nayak A., Gupta N., Pollard S., McAlary M., Taylor A.F., Rohane P. (2001). Treatment of childhood asthma with anti-immunoglobulin E antibody (omalizumab). Pediatrics.

[12] Buhl R., Soler M., Matz J., Townley R., O’Brien J., Noga O., Champain K., Fox H., Thirlwell J., Della Cioppa G. (2002). Omalizumab provides long-term control in patients with moderate-to-severe allergic asthma. Eur. Respir. J..

[13] Bousquet J., Cabrera P., Berkman N., Buhl R., Holgate S., Wenzel S., Fox H., Hedgecock S., Blogg M., Cioppa G.D. (2005). The effect of treatment with omalizumab, an anti-IgE antibody, on asthma exacerbations and emergency medical visits in patients with severe persistent asthma. Allergy.

[14] Holgate S.T., Djukanovic R., Casale T., Bousquet J. (2005). Anti-immunoglobulin E treatment with omalizumab in allergic diseases: An update on anti-inflammatory activity and clinical efficacy. Clin. Exp. Allergy.

[15] Metz M., Vadasz Z., Kocaturk E., Gimenez-Arnau A.M. (2020). Omalizumab Updosing in Chronic Spontaneous Urticaria: An Overview of Real-World Evidence. Clin. Rev. Allergy Immunol..

[16] Okudaira H., Komagata Y., Ogita T. (1980). T cell dependent and independent steps in IgE-B memory cell development. Int. Arch. Allergy Appl. Immunol..

[17] Nehls M., Pfeifer D., Schorpp M., Hedrich H., Boehm T. (1994). New member of the winged-helix protein family disrupted in mouse and rat nude mutations. Nature.

[18] Bulfone-Paus S., Bahri R. (2015). Mast Cells as Regulators of T Cell Responses. Front. Immunol..

[19] Kitamura Y., Go S., Hatanaka K. (1978). Decrease of mast cells in W/Wv mice and their increase by bone marrow transplantation. Blood.

[20] Kung T.T., Stelts D., Zurcher J.A., Jones H., Umland S.P., Kreutner W., Egan R.W., Chapman R.W. (1995). Mast cells modulate allergic pulmonary eosinophilia in mice. Am. J. Respir. Cell Mol. Biol..

[21] Nagai H., Yamaguchi S., Maeda Y., Tanaka H. (1996). Role of mast cells, eosinophils and IL-5 in the development of airway hyperresponsiveness in sensitized mice. Clin. Exp. Allergy.

[22] Takeda K., Hamelmann E., Joetham A., Shultz L.D., Larsen G.L., Irvin C.G., Gelfand E.W. (1997). Development of eosinophilic airway inflammation and airway hyperresponsiveness in mast cell-deficient mice. J. Exp. Med..

[23] Ogawa K., Kaminuma O., Kikkawa H., Nakata A., Asahina M., Egan R.W., Akiyama K., Mori A. (2002). Transient contribution of mast cells to pulmonary eosinophilia but not to hyper-responsiveness. Clin. Exp. Allergy.

[24] Komai M., Tanaka H., Masuda T., Nagao K., Ishizaki M., Sawada M., Nagai H. (2003). Role of Th2 responses in the development of allergen-induced airway remodelling in a murine model of allergic asthma. Br. J. Pharmacol..

[25] Iwamoto I., Tomoe S., Tomioka H., Takatsu K., Yoshida S. (1992). Role of CD4+ T lymphocytes and interleukin-5 in antigen-induced eosinophil recruitment into the site of cutaneous late-phase reaction in mice. J. Leukoc. Biol..

[26] Mukai K., Matsuoka K., Taya C., Suzuki H., Yokozeki H., Nishioka K., Hirokawa K., Etori M., Yamashita M., Kubota T. (2005). Basophils play a critical role in the development of IgE-mediated chronic allergic inflammation independently of T cells and mast cells. Immunity.

[27] Poddighe D., Mathias C.B., Brambilla I., Marseglia G.L., Oettgen H.C. (2018). Importance of Basophils in Eosinophilic Asthma: The Murine Counterpart. J. Biol. Reg. Homeos. Ag..

[28] Nishimura T., Saeki M., Kaminuma O., Matsuoka K., Yonekawa H., Mori A., Hiroi T. (2013). Existence of antigen-specific immunoglobulin E is not sufficient for allergic nasal eosinophil infiltration in mice. Int. Arch. Allergy Immunol..

[29] Kaminuma O., Mori A., Ogawa K., Nakata A., Kikkawa H., Naito K., Suko M., Okudaira H. (1997). Successful transfer of late phase eosinophil infiltration in the lung by infusion of helper T cell clones. Am. J. Respir. Cell Mol. Biol..

[30] Randolph D.A., Stephens R., Carruthers C.J., Chaplin D.D. (1999). Cooperation between Th1 and Th2 cells in a murine model of eosinophilic airway inflammation. J. Clin. Investig..

[31] Kaminuma O., Mori A. (2002). Potential initiation of eosinophilic skin inflammation by antigen-specific T helper type 2 cells. Int. Arch. Allergy Immunol..

[32] Nishimura T., Kaminuma O., Saeki M., Kitamura N., Matsuoka K., Yonekawa H., Mori A., Hiroi T. (2016). Essential Contribution of CD4+ T Cells to Antigen-Induced Nasal Hyperresponsiveness in Experimental Allergic Rhinitis. PLoS ONE.

[33] Watanabe N., Kaminuma O., Kitamura N., Hiroi T. (2016). Induced Treg Cells Augment the Th17-Mediated Intestinal Inflammatory Response in a CTLA4-Dependent Manner. PLoS ONE.

[34] Robinson D.S., Hamid Q., Ying S., Tsicopoulos A., Barkans J., Bentley A.M., Corrigan C., Durham S.R., Kay A.B. (1992). Predominant TH2-like bronchoalveolar T-lymphocyte population in atopic asthma. N. Eng. J. Med..

[35] van Reijsen F.C., Bruijnzeel-Koomen C.A., Kalthoff F.S., Maggi E., Romagnani S., Westland J.K., Mudde G.C. (1992). Skin-derived aeroallergen-specific T-cell clones of Th2 phenotype in patients with atopic dermatitis. J. Allergy Clin. Immunol..

[36] Bousquet J., Chanez P., Lacoste J.Y., Barneon G., Ghavanian N., Enander I., Venge P., Ahlstedt S., Simony-Lafontaine J., Godard P. (1990). Eosinophilic inflammation in asthma. N. Eng. J. Med..

[37] Kay A.B. (1991). Asthma and inflammation. J. Allergy Clin. Immunol..

[38] Bochner B.S., Undem B.J., Lichtenstein L.M. (1994). Immunological aspects of allergic asthma. Annu. Rev. Immunol..

[39] Wills-Karp M. (1999). Immunologic basis of antigen-induced airway hyperresponsiveness. Annu. Rev. Immunol..

[40] Garcia G., Taille C., Laveneziana P., Bourdin A., Chanez P., Humbert M. (2013). Anti-interleukin-5 therapy in severe asthma. Eur. Respir. Rev..

[41] Price D.B., Rigazio A., Campbell J.D., Bleecker E.R., Corrigan C.J., Thomas M., Wenzel S.E., Wilson A.M., Small M.B., Gopalan G. (2015). Blood eosinophil count and prospective annual asthma disease burden: A UK cohort study. Lancet Respir. Med..

[42] McBrien C.N., Menzies-Gow A. (2017). The Biology of Eosinophils and Their Role in Asthma. Front. Med. (Lausanne).

[43] Walker C., Bode E., Boer L., Hansel T.T., Blaser K., Virchow J.C. (1992). Allergic and nonallergic asthmatics have distinct patterns of T-cell activation and cytokine production in peripheral blood and bronchoalveolar lavage. Am. Rev. Respir. Dis..

[44] Walker C., Bauer W., Braun R.K., Menz G., Braun P., Schwarz F., Hansel T.T., Villiger B. (1994). Activated T cells and cytokines in bronchoalveolar lavages from patients with various lung diseases associated with eosinophilia. Am. J. Respir. Crit. Care Med..

[45] Truyen E., Coteur L., Dilissen E., Overbergh L., Dupont L.J., Ceuppens J.L., Bullens D.M. (2006). Evaluation of airway inflammation by quantitative Th1/Th2 cytokine mRNA measurement in sputum of asthma patients. Thorax.

[46] Mori A., Suko M., Nishizaki Y., Kaminuma O., Kobayashi S., Matsuzaki G., Yamamoto K., Ito K., Tsuruoka N., Okudaira H. (1995). IL-5 production by CD4+ T cells of asthmatic patients is suppressed by glucocorticoids and the immunosuppressants FK506 and cyclosporin A. Int. Immunol..

[47] Nakata A., Kaminuma O., Ogawa K., Fujimura H., Fushimi K., Kikkawa H., Naito K., Ikezawa K., Egan R.W., Mori A. (2001). Correlation between eosinophilia induced by CD4(+) T cells and bronchial hyper-responsiveness. Int. Immunol..

[48] Humbles A.A., Lloyd C.M., McMillan S.J., Friend D.S., Xanthou G., McKenna E.E., Ghiran S., Gerard N.P., Yu C., Orkin S.H. (2004). A critical role for eosinophils in allergic airways remodeling. Science.

[49] Lee J.J., Dimina D., Macias M.P., Ochkur S.I., McGarry M.P., O’Neill K.R., Protheroe C., Pero R., Nguyen T., Cormier S.A. (2004). Defining a link with asthma in mice congenitally deficient in eosinophils. Science.

[50] Saeki M., Kaminuma O., Nishimura T., Kitamura N., Mori A., Hiroi T. (2016). Th9 cells elicit eosinophil-independent bronchial hyperresponsiveness in mice. Allergol. Int..

[51] Li X.M., Chopra R.K., Chou T.Y., Schofield B.H., Wills-Karp M., Huang S.K. (1996). Mucosal IFN-gamma gene transfer inhibits pulmonary allergic responses in mice. J. Immunol..

[52] Cohn L., Homer R.J., Niu N., Bottomly K. (1999). T helper 1 cells and interferon gamma regulate allergic airway inflammation and mucus production. J. Exp. Med..

[53] Hansen G., Berry G., DeKruyff R.H., Umetsu D.T. (1999). Allergen-specific Th1 cells fail to counterbalance Th2 cell-induced airway hyperreactivity but cause severe airway inflammation. J. Clin. Investig..

[54] Dardalhon V., Awasthi A., Kwon H., Galileos G., Gao W., Sobel R.A., Mitsdoerffer M., Strom T.B., Elyaman W., Ho I.C. (2008). IL-4 inhibits TGF-beta-induced Foxp3+ T cells and, together with TGF-beta, generates IL-9+ IL-10+ Foxp3(-) effector T cells. Nat. Immunol..

[55] Veldhoen M., Uyttenhove C., van Snick J., Helmby H., Westendorf A., Buer J., Martin B., Wilhelm C., Stockinger B. (2008). Transforming growth factor-beta ‘reprograms’ the differentiation of T helper 2 cells and promotes an interleukin 9-producing subset. Nat. Immunol..

[56] Micosse C., von Meyenn L., Steck O., Kipfer E., Adam C., Simillion C., Seyed Jafari S.M., Olah P., Yawlkar N., Simon D. (2019). Human “TH9” cells are a subpopulation of PPAR-gamma(+) TH2 cells. Sci. Immunol..

[57] Harrington L.E., Hatton R.D., Mangan P.R., Turner H., Murphy T.L., Murphy K.M., Weaver C.T. (2005). Interleukin 17-producing CD4+ effector T cells develop via a lineage distinct from the T helper type 1 and 2 lineages. Nat. Immunol..

[58] Langrish C.L., Chen Y., Blumenschein W.M., Mattson J., Basham B., Sedgwick J.D., McClanahan T., Kastelein R.A., Cua D.J. (2005). IL-23 drives a pathogenic T cell population that induces autoimmune inflammation. J. Exp. Med..

[59] Liang S.C., Tan X.Y., Luxenberg D.P., Karim R., Dunussi-Joannopoulos K., Collins M., Fouser L.A. (2006). Interleukin (IL)-22 and IL-17 are coexpressed by Th17 cells and cooperatively enhance expression of antimicrobial peptides. J. Exp. Med..

[60] Eyerich S., Eyerich K., Pennino D., Carbone T., Nasorri F., Pallotta S., Cianfarani F., Odorisio T., Traidl-Hoffmann C., Behrendt H. (2009). Th22 cells represent a distinct human T cell subset involved in epidermal immunity and remodeling. J. Clin. Investig..

[61] Tato C.M., Laurence A., O’Shea J.J. (2006). Helper T cell differentiation enters a new era: Le roi est mort; vive le roi!. J. Exp. Med..

[62] Szegedi K., van Lier A., Res P.C., Chielie S., Bos J.D., Kezic S., Middelkamp-Hup M.A., Luiten R.M. (2018). House dust mite allergens Der f and Der p induce IL-31 production by blood-derived T cells from atopic dermatitis patients. Exp. Dermatol..

[63] Suarez-Alvarez B., Rodriguez R.M., Fraga M.F., Lopez-Larrea C. (2012). DNA methylation: A promising landscape for immune system-related diseases. Trends. Genet..

[64] Potaczek D.P., Harb H., Michel S., Alhamwe B.A., Renz H., Tost J. (2017). Epigenetics and allergy: From basic mechanisms to clinical applications. Epigenomics.

[65] McKinley L., Alcorn J.F., Peterson A., Dupont R.B., Kapadia S., Logar A., Henry A., Irvin C.G., Piganelli J.D., Ray A. (2008). TH17 cells mediate steroid-resistant airway inflammation and airway hyperresponsiveness in mice. J. Immunol..

[66] Kaminuma O., Ohtomo T., Mori A., Nagakubo D., Hieshima K., Ohmachi Y., Noda Y., Katayama K., Suzuki K., Motoi Y. (2012). Selective down-regulation of Th2 cell-mediated airway inflammation in mice by pharmacological intervention of CCR4. Clin. Exp. Allergy.

[67] Saeki M., Kaminuma O., Nishimura T., Kitamura N., Mori A., Hiroi T. (2017). Th9 cells induce steroid-resistant bronchial hyperresponsiveness in mice. Allergol. Int..

[68] Ying S., Durham S.R., Corrigan C.J., Hamid Q., Kay A.B. (1995). Phenotype of cells expressing mRNA for TH2-type (interleukin 4 and interleukin 5) and TH1-type (interleukin 2 and interferon gamma) cytokines in bronchoalveolar lavage and bronchial biopsies from atopic asthmatic and normal control subjects. Am. J. Respir. Cell Mol. Biol..

[69] Pene J., Chevalier S., Preisser L., Venereau E., Guilleux M.H., Ghannam S., Moles J.P., Danger Y., Ravon E., Lesaux S. (2008). Chronically inflamed human tissues are infiltrated by highly differentiated Th17 lymphocytes. J. Immunol..

[70] Hoppenot D., Malakauskas K., Lavinskiene S., Bajoriuniene I., Kalinauskaite V., Sakalauskas R. (2015). Peripheral blood Th9 cells and eosinophil apoptosis in asthma patients. Medicina (Kaunas).

[71] Jia L., Wang Y., Li J., Li S., Zhang Y., Shen J., Tan W., Wu C. (2017). Detection of IL-9 producing T cells in the PBMCs of allergic asthmatic patients. BMC Immunol..

[72] Murdaca G., Colombo B.M., Puppo F. (2011). The role of Th17 lymphocytes in the autoimmune and chronic inflammatory diseases. Intern. Emerg. Med..

[73] Yu Q.L., Chen Z. (2018). Establishment of different experimental asthma models in mice. Exp. Ther. Med..

[74] Tan H.T., Hagner S., Ruchti F., Radzikowska U., Tan G., Altunbulakli C., Eljaszewicz A., Moniuszko M., Akdis M., Akdis C.A. (2019). Tight junction, mucin, and inflammasome-related molecules are differentially expressed in eosinophilic, mixed, and neutrophilic experimental asthma in mice. Allergy.

[75] Sakaguchi S., Sakaguchi N., Asano M., Itoh M., Toda M. (1995). Immunologic self-tolerance maintained by activated T cells expressing IL-2 receptor alpha-chains (CD25). Breakdown of a single mechanism of self-tolerance causes various autoimmune diseases. J. Immunol..

[76] Palomares O., Akdis M., Martin-Fontecha M., Akdis C.A. (2017). Mechanisms of immune regulation in allergic diseases: The role of regulatory T and B cells. Immunol. Rev..

[77] Lambrecht B.N., Hammad H. (2015). The immunology of asthma. Nat. Immunol..

[78] Gandhi N.A., Bennett B.L., Graham N.M., Pirozzi G., Stahl N., Yancopoulos G.D. (2016). Targeting key proximal drivers of type 2 inflammation in disease. Nat. Rev. Drug. Discov..

[79] Miethe S., Guarino M., Alhamdan F., Simon H.U., Renz H., Dufour J.F., Potaczek D.P., Garn H. (2018). Effects of obesity on asthma: Immunometabolic links. Pol. Arch. Intern. Med..

[80] Jonckheere A.C., Bullens D.M.A., Seys S.F. (2019). Innate lymphoid cells in asthma: Pathophysiological insights from murine models to human asthma phenotypes. Curr. Opin. Allergy Clin. Immunol..

[81] Potaczek D.P., Miethe S., Schindler V., Alhamdan F., Garn H. (2020). Role of airway epithelial cells in the development of different asthma phenotypes. Cell Signal.

[82] Akdis C.A., Bachert C., Cingi C., Dykewicz M.S., Hellings P.W., Naclerio R.M., Schleimer R.P., Ledford D. (2013). Endotypes and phenotypes of chronic rhinosinusitis: A PRACTALL document of the European Academy of Allergy and Clinical Immunology and the American Academy of Allergy, Asthma & Immunology. J. Allergy Clin. Immunol..

[83] Agache I., Akdis C.A. (2016). Endotypes of allergic diseases and asthma: An important step in building blocks for the future of precision medicine. Allergol. Int..

[84] Barnden M.J., Allison J., Heath W.R., Carbone F.R. (1998). Defective TCR expression in transgenic mice constructed using cDNA-based alpha- and beta-chain genes under the control of heterologous regulatory elements. Immunol. Cell Biol..

[85] Murphy K.M., Heimberger A.B., Loh D.Y. (1990). Induction by antigen of intrathymic apoptosis of CD4+CD8+TCRlo thymocytes in vivo. Science.

[86] Nishimura T., Saeki M., Motoi Y., Kitamura N., Mori A., Kaminuma O., Hiroi T. (2014). Selective suppression of Th2 cell-mediated lung eosinophilic inflammation by anti-major facilitator super family domain containing 10 monoclonal antibody. Allergol. Int..

[87] Kaminuma O., Nishimura T., Kitamura N., Saeki M., Hiroi T., Mori A. (2018). T-Helper Type 2 Cells Direct Antigen-Induced Eosinophilic Skin Inflammation in Mice. Allergy Asthma Immunol. Res..

[88] Sato T., Sasahara T., Nakamura Y., Osaki T., Hasegawa T., Tadakuma T., Arata Y., Kumagai Y., Katsuki M., Habu S. (1994). Naive T cells can mediate delayed-type hypersensitivity response in T cell receptor transgenic mice. Eur. J. Immunol..

[89] Nakajima-Adachi H., Ebihara A., Kikuchi A., Ishida T., Sasaki K., Hirano K., Watanabe H., Asai K., Takahashi Y., Kanamori Y. (2006). Food antigen causes TH2-dependent enteropathy followed by tissue repair in T-cell receptor transgenic mice. J. Allergy Clin. Immunol..

[90] Nakajima-Adachi H., Kikuchi A., Fujimura Y., Shibahara K., Makino T., Goseki-Sone M., Kihara-Fujioka M., Nochi T., Kurashima Y., Igarashi O. (2014). Peyer’s patches and mesenteric lymph nodes cooperatively promote enteropathy in a mouse model of food allergy. PLoS ONE.

[91] Nakajima-Adachi H., Koike E., Totsuka M., Hiraide E., Wakatsuki Y., Kiyono H., Hachimura S. (2012). Two distinct epitopes on the ovalbumin 323-339 peptide differentiating CD4(+)T cells into the Th2 or Th1 phenotype. Biosci. Biotechnol. Biochem..

[92] Jarman E.R., Tan K.A., Lamb J.R. (2005). Transgenic mice expressing the T cell antigen receptor specific for an immunodominant epitope of a major allergen of house dust mite develop an asthmatic phenotype on exposure of the airways to allergen. Clin. Exp. Allergy.

[93] Lemaire M.M., Dumoutier L., Warnier G., Uyttenhove C., Van Snick J., de Heusch M., Stevens M., Renauld J.C. (2011). Dual TCR expression biases lung inflammation in DO11.10 transgenic mice and promotes neutrophilia via microbiota-induced Th17 differentiation. J. Immunol..

[94] Constant S., Pfeiffer C., Woodard A., Pasqualini T., Bottomly K. (1995). Extent of T cell receptor ligation can determine the functional differentiation of naive CD4+ T cells. J. Exp. Med..

[95] Hosken N.A., Shibuya K., Heath A.W., Murphy K.M., O’Garra A. (1995). The effect of antigen dose on CD4+ T helper cell phenotype development in a T cell receptor-alpha beta-transgenic model. J. Exp. Med..

[96] Robinson J., Barker D.J., Georgiou X., Cooper M.A., Flicek P., Marsh S.G.E. (2020). IPD-IMGT/HLA Database. Nucleic Acids Res..

[97] Wilmut I., Schnieke A.E., McWhir J., Kind A.J., Campbell K.H. (1997). Viable offspring derived from fetal and adult mammalian cells. Nature.

[98] Wakayama T., Perry A.C., Zuccotti M., Johnson K.R., Yanagimachi R. (1998). Full-term development of mice from enucleated oocytes injected with cumulus cell nuclei. Nature.

[99] Hochedlinger K., Jaenisch R. (2002). Monoclonal mice generated by nuclear transfer from mature B and T donor cells. Nature.

[100] Serwold T., Hochedlinger K., Inlay M.A., Jaenisch R., Weissman I.L. (2007). Early TCR expression and aberrant T cell development in mice with endogenous prerearranged T cell receptor genes. J. Immunol..

[101] Kirak O., Frickel E.M., Grotenbreg G.M., Suh H., Jaenisch R., Ploegh H.L. (2010). Transnuclear mice with predefined T cell receptor specificities against Toxoplasma gondii obtained via SCNT. Science.

[102] Inoue K., Wakao H., Ogonuki N., Miki H., Seino K., Nambu-Wakao R., Noda S., Miyoshi H., Koseki H., Taniguchi M. (2005). Generation of cloned mice by direct nuclear transfer from natural killer T cells. Curr. Biol..

[103] Kamimura S., Inoue K., Ogonuki N., Hirose M., Oikawa M., Yo M., Ohara O., Miyoshi H., Ogura A. (2013). Mouse cloning using a drop of peripheral blood. Biol. Reprod..

[104] Eggan K., Akutsu H., Loring J., Jackson-Grusby L., Klemm M., Rideout W.M., Yanagimachi R., Jaenisch R. (2001). Hybrid vigor, fetal overgrowth, and viability of mice derived by nuclear cloning and tetraploid embryo complementation. Proc. Natl. Acad. Sci. USA.

[105] Ogura A., Inoue K., Wakayama T. (2013). Recent advancements in cloning by somatic cell nuclear transfer. Philos. Trans. R. Soc. Lond. B Biol. Sci..

[106] Kaminuma O., Katayama K., Inoue K., Saeki M., Nishimura T., Kitamura N., Shimo Y., Tofukuji S., Ishida S., Ogonuki N. (2017). Hyper-reactive cloned mice generated by direct nuclear transfer of antigen-specific CD4(+) T cells. EMBO Rep..

[107] Tubo N.J., Pagan A.J., Taylor J.J., Nelson R.W., Linehan J.L., Ertelt J.M., Huseby E.S., Way S.S., Jenkins M.K. (2013). Single naive CD4+ T cells from a diverse repertoire produce different effector cell types during infection. Cell.

[108] Johnson J.R., Wiley R.E., Fattouh R., Swirski F.K., Gajewska B.U., Coyle A.J., Gutierrez-Ramos J.C., Ellis R., Inman M.D., Jordana M. (2004). Continuous exposure to house dust mite elicits chronic airway inflammation and structural remodeling. Am. J. Respir. Crit. Care Med..

[109] Ulrich K., Hincks J.S., Walsh R., Wetterstrand E.M., Fidock M.D., Sreckovic S., Lamb D.J., Douglas G.J., Yeadon M., Perros-Huguet C. (2008). Anti-inflammatory modulation of chronic airway inflammation in the murine house dust mite model. Pulm. Pharmacol. Ther..

[110] Shimizu H., Obase Y., Katoh S., Mouri K., Kobashi Y., Oka M. (2013). Critical role of interleukin-5 in the development of a mite antigen-induced chronic bronchial asthma model. Inflamm. Res..

